# Common predictors of cervical cancer related mortality in Ethiopia. A systematic review and meta-analysis

**DOI:** 10.1186/s12889-024-18238-x

**Published:** 2024-03-19

**Authors:** Hunduma Dina Hambisa, Berhane Teklay Asfaha, Biniam Ambisa, Abebech Gudeta Beyisho

**Affiliations:** 1 Department of Midwifery, School of Nursing and Midwifery, Institutes of Health Science, Wollega University, Nekemte, Ethiopia; 2 Department of Public Health, College of Health science, Assosa University, Assosa, Ethiopia; 3 Department of Midwifery, College of Health science, Assosa University, Assosa, Ethiopia

**Keywords:** Cervical cancer, Magnitude, Predictors, Systematic review, Meta-analysis

## Abstract

**Background:**

Cervical cancer accounts for 7.5% of all female cancer related deaths worldwide; peaking between the ages of 35 and 65, and not only kills young women but also destroys families with young children.

**Objective:**

This review was intended to measure national level magnitude and the most common predictors of cervical cancer related mortality in Ethiopia.

**Methods:**

Common Public databases like Science Direct, Embase, the Cochrane Library, and PubMed were thoroughly searched. The STATA 14 and Rev-Manager 5.3 statistical software packages were used for analysis, as well as a standardized data abstraction tool created in Microsoft Excel. The Cochrane Q-test statistics and the I^2^ test were used to assess non-uniformity. The pooled magnitude and predictors of cervical cancer related mortality were estimated using fixed-effect and random-effect models, respectively.

**Result:**

The pooled mortality among cervical cancer patients was estimated that 16.39% at 95% confidence level fall in 13.89–18.88% in Ethiopia. The most common predictors of cervical cancer related mortality were late diagnosed, radiation therapy alone, and Being anemic were identified by this review. Among cervical cancer treatment modalities effectiveness of surgery with adjuvant therapy was also approved in this meta-analysis.

**Conclusion and recommendation:**

In this study high cervical cancer-related mortality was reported as compared to national strategies to alleviate cervical cancer related mortality. Advanced implementation of cervical cancer screening at the national level for early diagnosis, anaemia detection, and combination anticancer therapy during initiation, as well as combination therapy, is critical to improve cervical cancer patient survival and decreasing mortality rates.

**Supplementary Information:**

The online version contains supplementary material available at 10.1186/s12889-024-18238-x.

## Background

In 2018, there were an estimated 570,000 cases and 311,000 deaths from cervical cancer worldwide, making it one of the top four common causes of cancer-related mortality among women [1]. Worldwide cancer-related death figures show that developed countries accounted for 85% of cervical cancer mortality, with low- and middle-income countries having an 18-fold higher death rate from the disease [2]. Cervical cancer is caused by oncogenic subtypes of the human papillomavirus [3]. The disease that kills young women and destroys families with small children also causes 7.5% of cancer-related deaths among women globally, with a peak incidence between the ages of 35 and 65 [4, 5].

As a result of population ageing and growth, as well as an increase in the prevalence of risk factors associated with economic change, such as smoking, obesity, physical inactivity, and reproductive behaviors, cancer is becoming more prevalent in Africa [6]. In Eastern, Western, Middle, and Southern Africa, women die from cancer mostly from cervical cancer [7]. Cervical cancer is expected to kill over 443,000 women worldwide by 2030, the vast majority of whom will be in Sub-Saharan Africa [8].

Each year, approximately 6,300 new cases of cervical cancer are diagnosed in Ethiopia cancer treatment centers, and approximately 4,884 women die from the disease [9]. According to data from TikurAnbesa Specialized Hospital (TASH) radiotherapy center, cervical cancer is the second most common type of cancer among patients attending the oncology center [10]. According to various sources, treatment options include surgery, chemotherapy, radiotherapy, or a combination of the three [11–13].

There is no study which shows country level of magnitude of cervical cancer related death and predictors of this mortality irrespective of tremendous updated cervical cancer therapies. Thus, this study was designed to measure overall magnitudes of cervical cancer related mortality and its predictors.

Conducting a review study as a strategy evaluation is so mandatory. Additionally, there is no finding that has been used to raise public awareness about predictors of death among cervical cancer patients, which paves the way for preventive and curative approaches for early detection and proper treatment.

## Methods

### Study protocol

**Fig. 1:**
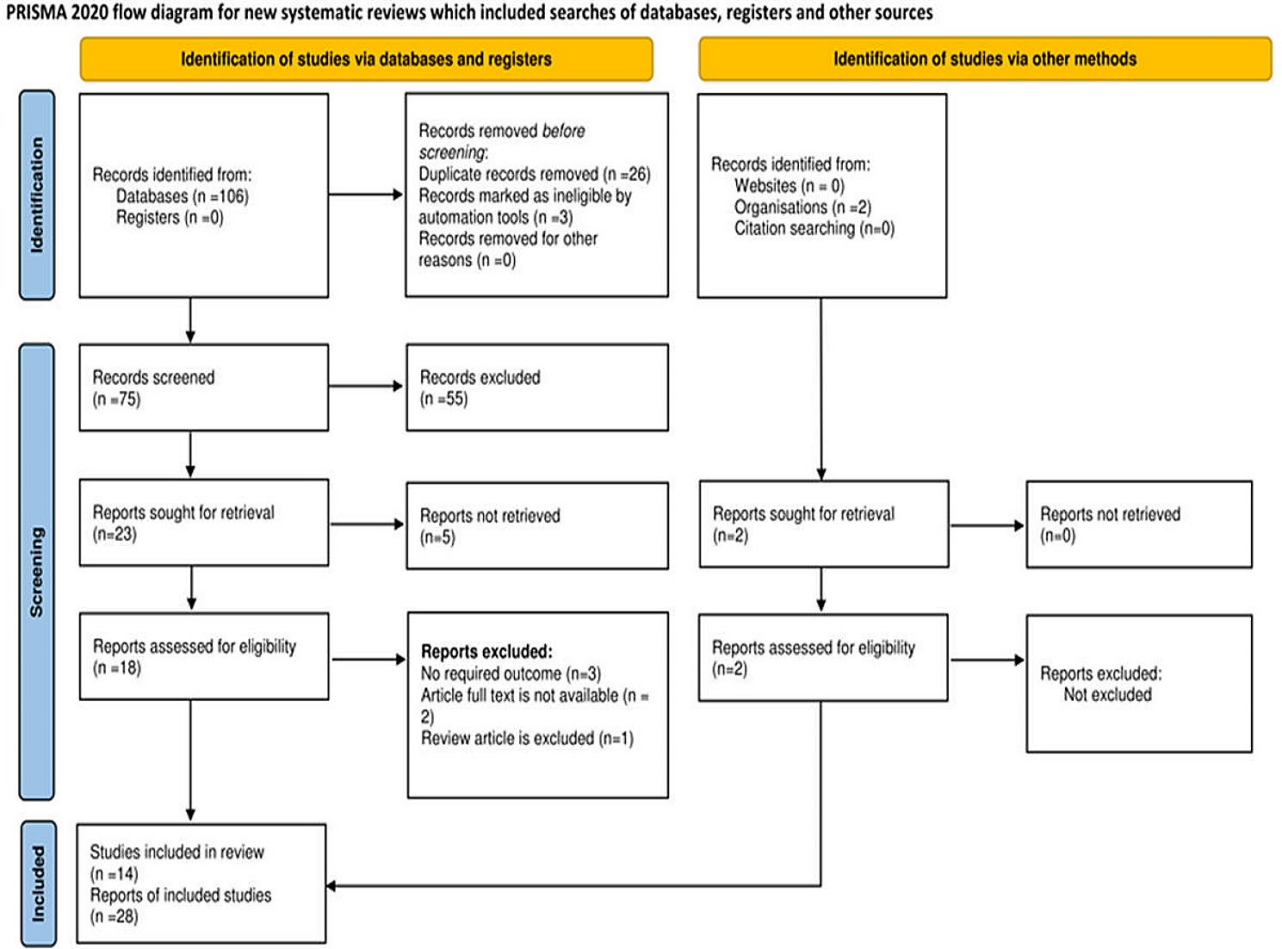
PIRSMA flowchart diagram of included article

This study’s protocol was based on the PRISMA recommendations for systematic reviews and meta-analyses [14]. Three distinct researchers worked independently to finish each step. The screening was based on methodological issues, incomplete data, or full text that was not accessible from the analysis [15]. The fourth researcher resolved any disagreements by examining whether the results were consistent. Additionally articles published in English online from university repositories were also considered. Then, described by Preferred Reporting Items for Systematic Review and Meta-analysis protocol (PRISMA-P) (fig. 1). (Additional file 1)

### Objective of the study

- To determine the national-level pooled magnitude cervical cancer related mortality.
- To identify predictors associated with cervical cancer related mortality.
- To detect heterogeneity between the studies at the national level.

### Sources of data and ways of search

Common Public databases like Articles conducted on pediatrics virological failure were searched in Google Scholar, PubMed, Embase, and Cochrane Library search engine using different Medical Subject Heading (MeSH). The search terms were used individually as well as in conjunction with Boolean operators such as “OR” and “AND.” Citations discovered using our search terms were imported into EndNote-X6 software, and duplicate articles were removed. (Additional file 2).

### Inclusion and exclusion criteria

The review attempted to include published studies with epidemiological data on cervical cancer mortality and predictors conducted. The PICOS evidence-based medicine framework served as our basis for inclusion criteria, and nonrandomized studies met the following PICOS-standardized components [16].

PICO (population, intervention, comparator and outcome) of the study.

P: Cervical cancer patients.

I: Categories of variables those are positively associated with virological failure.

C: Categories of variables those are significantly associated with PPD.

O: Pediatrics with HIV infection and on ART experienced virological failure.

The following points were used as exclusion criteria.

1. Studies with sample sizes with cervical cancer and other comorbidities..
2. Studies included more than 18-year-old participants..
3. Studies that reported immunological and mixed measurements..
4. Studies that reported un-operationalized out come..
5. Complete data, i.e., lack of variable of interest..

### Assessment of quality of included studies

Newcastle-Ottawa Scale assessment of Critical Appraisal Checklist for non-randomized Studies was used to determined quality status of the article and included articles were accepted at the level of above mean score out of 14 overall score [17]. (Additional file 3)

### Measurements of outcome

Death among cervical cancer was considered as cervical cancer related mortality and Predictors were factors those consistently with mortality in more than or four articles were considered in this meta- analysis.

### Characteristics of included studies

This meta-analysis included 14 studies that reported on the magnitude and predictors of mortality among cervical cancer patients in Ethiopia. Totally, 9,260 cervical cancer patients were included in the study. Articles published between January 2013 and March 30, 2023 was taken into account. This study looked at the most common predictors of mortality in cervical cancer patients. Those predictors were, late diagnosis [18–21], radiation therapy only [10, 22, 23], surgery with adjuvant therapy [24–26] and anemic status of the patient [27–29] were selected based on their frequency identified as the predictors by different studies (Table 1).

**Table 1:** Summary of studies involved in meta-analysis

Author Name	Publication year	Study Design	Total Sample	Total Events (%)	Response rate (%)	Quality score
Aguade et al.	2023	Cohort	322	48	98.0	12
Argefa et al.	2022	cohort	175	33	91.0	13
Begoihn et al.	2019	cohort	1495	52	95.0	11
Deressa et al.	2021	cohort	242	57.9	89.0	13
Fikreaddis T et al.	2018	Cross-sectional	252	5.6	98.5	9
Gashu et al.	2023	cohort	322	36.60	92.0	13
Gizaw et al.	2017	cohort	1655	52	89.0	12
Gurmu M et al.	2018	Cohort	907	38.5	90.0	12
Kantelhardt et al.	2014	cohort	1,059	23.5	98.0	11
Mebratie et al.	2022	cohort	422	27.66	89.0	13
Mölle G et al.	2016	cohort	1009	49	92.0	11
Olyad M et al.	2021	cohort	418	9.9	91	8
Seifu et al.	2022	Cohort	348	31	99.4	12
Wassie et al.	2019	cohort	634	38.62	98.0	12

### Data extraction techniques

The necessary data was taken from a predetermined set of articles using a Microsoft Excel template. The name of the author, the year of publication, the location, the age range, the sample size, the response rate, the number of study subjects with the desired outcome, the prevalence rates, and the predictors significantly associated with death from cervical cancer were all contained in a Microsoft Excel spreadsheet. (Additional file 4 and Additional file 5)

### Data synthesis and analysis

The heterogeneity inverse variance (I^2^) test was used to assess study non-uniformity, and Cochran’s Q test (*P*-value less than 0.1) revealed statistically significant results [30].. The I^2^ value ranges from 0 to 100%. I^2^ 75% indicates that there is significant heterogeneity across studies, with a *P*-value of 0.05 used to declare significant heterogeneity [31]. The pooled size magnitude of cervical cancer related mortality was estimated using a random effect model because I^2^ = 78.50 at *p* = 0.00, indicating the presence of heterogeneity between studies. Furthermore, we used a random model for subgroup analysis by identified predictors to deal with the significance of their significance in cervical cancer-related mortality at I^2^ = 96.4%, indicating high heterogeneity across studies [32].

### Publication bias assessment

**Fig. 2:**
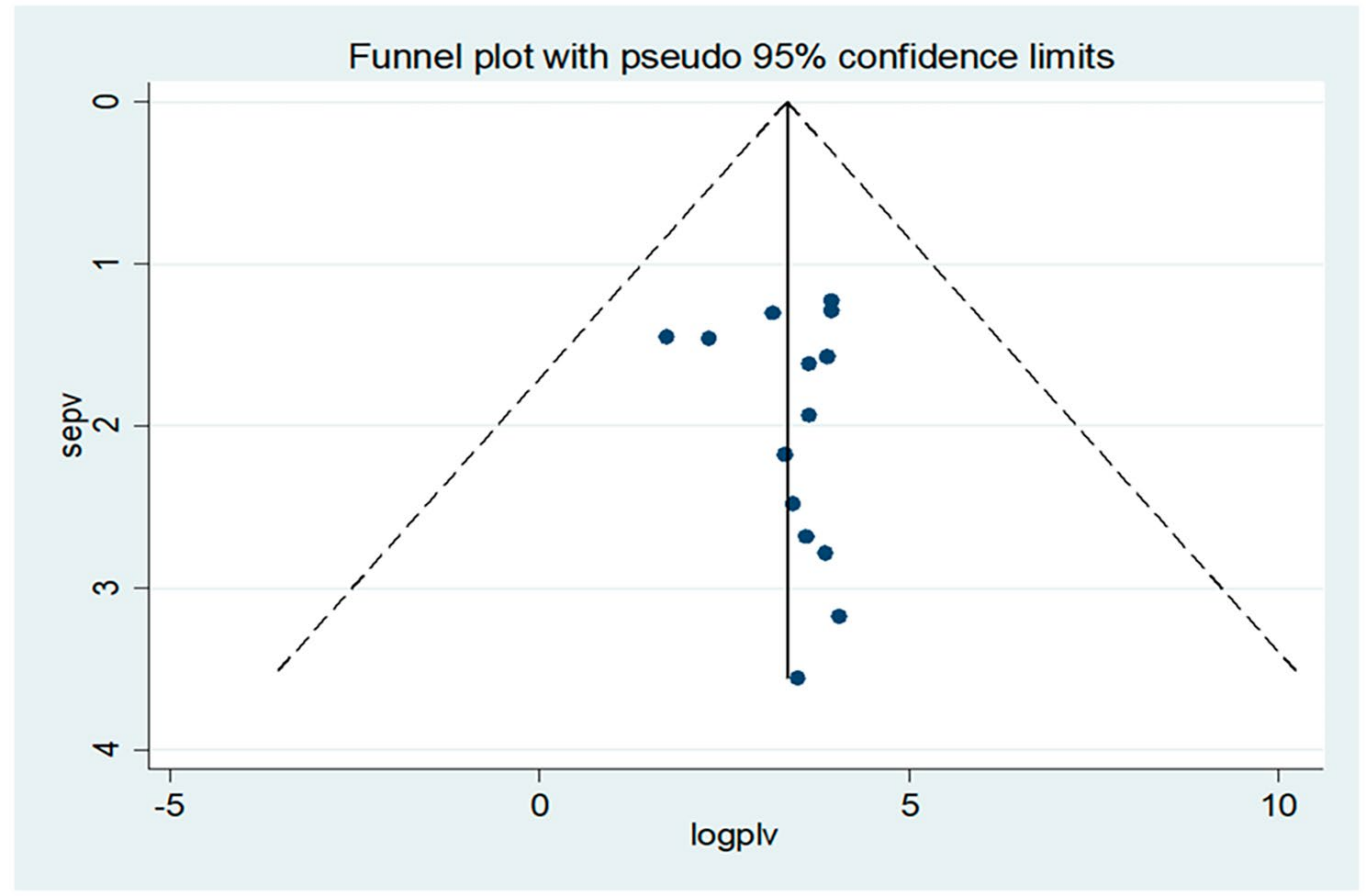
Funnel plot for test of publication bias

A funnelplot graph was used visually to assess publication bias. The symmetry of the funnel plot revealed that there was no potential publication bias [33] (Fig. 2). Egger’s and Begg’s tests were also run at the 5% significant level; the distribution of each study, as well as a *P*-value greater or less than 0.05, were used to determine the presence or absence of publication bias [30] (Table 2**)**.

**Table 2:** Egger’s test for small study effect

Number of studies = 14				Root MSE = 10.49	
**Stf_Eff**	**Coefficient**	**Std_error**	**t-value**	**p>|t|**	**95% confidence Interval**
Slope	31.33	15.75	1.99	0.070	-2.99 65.65
Bias	2.06	9.09	0.23	0.043	-17.76 21.88

Test of H0: Slightly small study effects detected *P* = 0.043

The Egger’s test output also indicates that there is no publication bias at (*P* = 0.825). However, the result of Begg’s test was notable in the presence of minimal publication bias (*P* = 0.047). Trim and fill analysis was thus performed following pooled effect analysis (Fig. 2**)**

## Result

### Pooled effect size of mortality among cervical cancer patients in Ethiopia

**Fig. 3:**
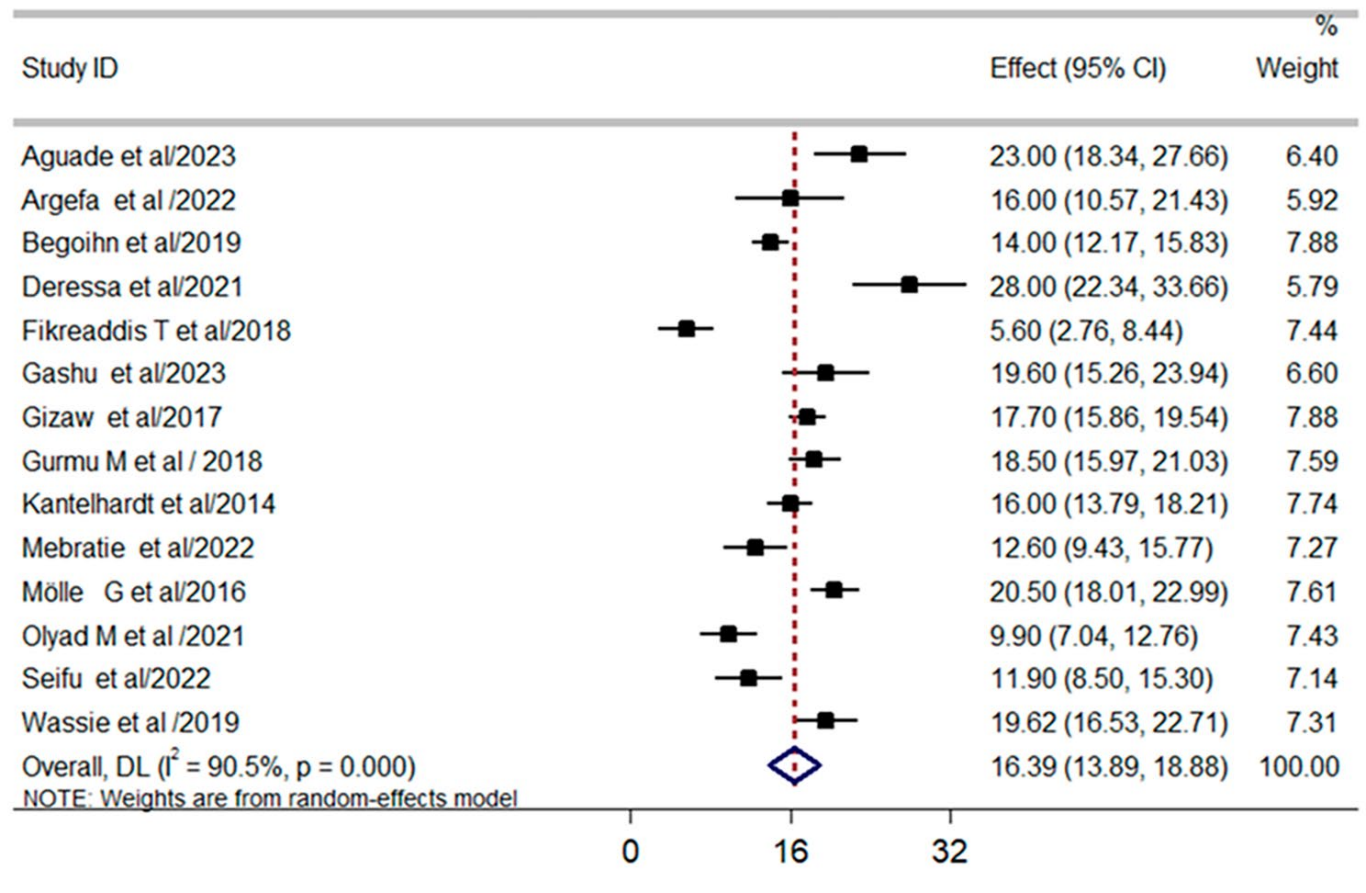
Forest plot showing pooled effect size of cervical cancer in Ethiopia

The pooled mortality among Ethiopian cervical cancer patients was 16.39% (95% confidence level; 13.89-18.88%). Because of heterogeneity was observed between the included primary studies at I^2^ = 90.50% and *P* = 0.00, we used random effect model to compute the pooled effect size of mortality among cervical cancer patients as described by figure of forest plot below (Fig. 3).

### Regression analysis of study uniformity

Meta-regression was used to identify the source of non-uniformity, and *p*-values greater than 0.1 revealed that there was no statistically significant non-uniformity between the studies (Table 3).

**Table 3:** Regression analysis of sample size influence between study non-uniformity

Number of studies = 16		Root MSE = 0.2841			
**Stf_Eff**	**Coefficient**	**Std_error**	**t-value**	**p>|t|**	**95% confidence Interval**
slope	2.796989	0.4632279	6.04	0.000	1.803464 3.790514
bias	− 0.0815417	0.2849218	-0.29	0.779	− 0.6926382 0.5295548

Test of H0: no small-study effects *P* = 0.779

**Fig. 4:**
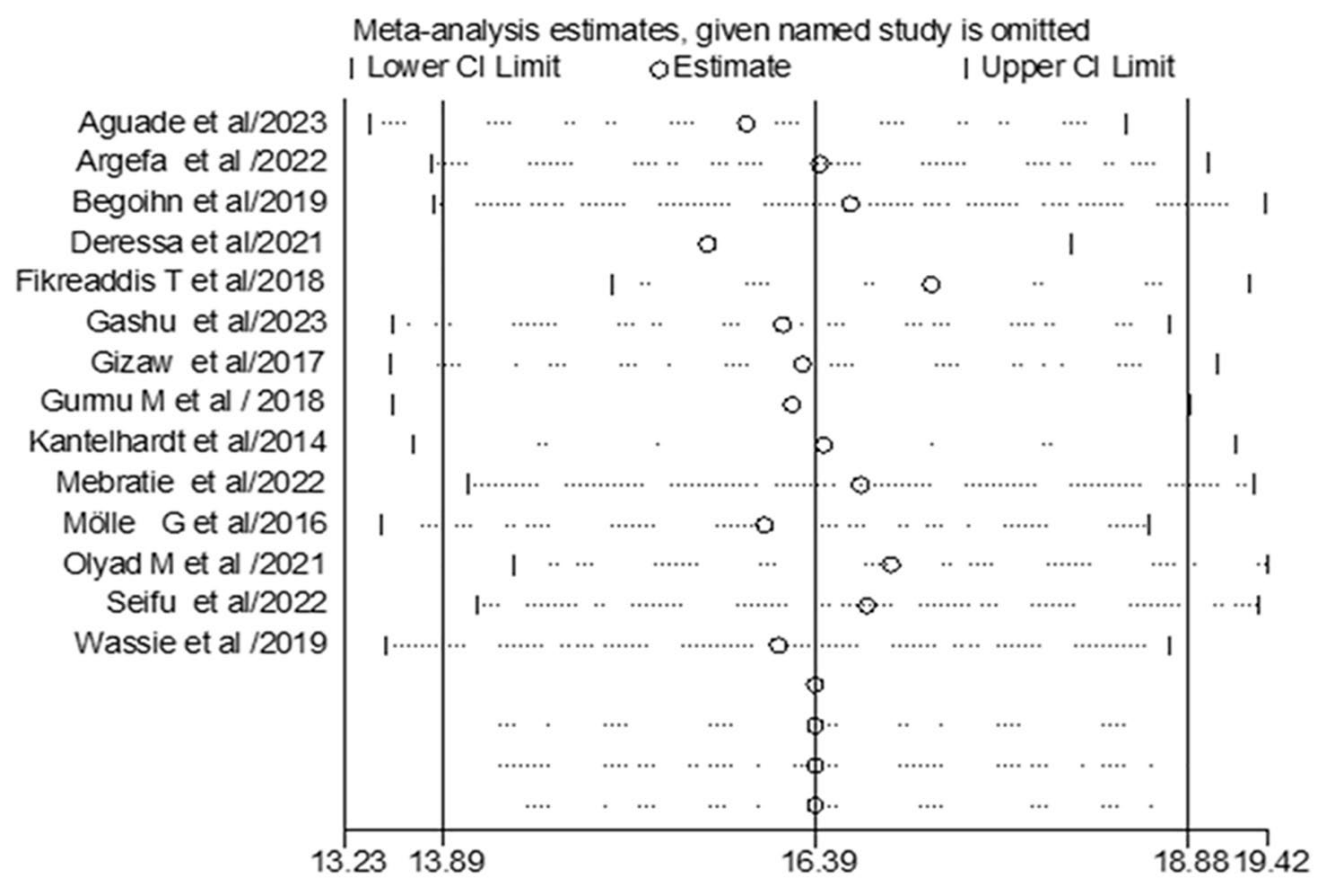
Sensitivity analysis of included studies

A sensitivity analysis was also performed to determine the effect size of each study on the pooled effect size. Two investigators carried out the statistical analysis independently, and the results were cross-checked for reliability, (Fig. 4).

### Trim and fill analysis

**Fig. 5:**
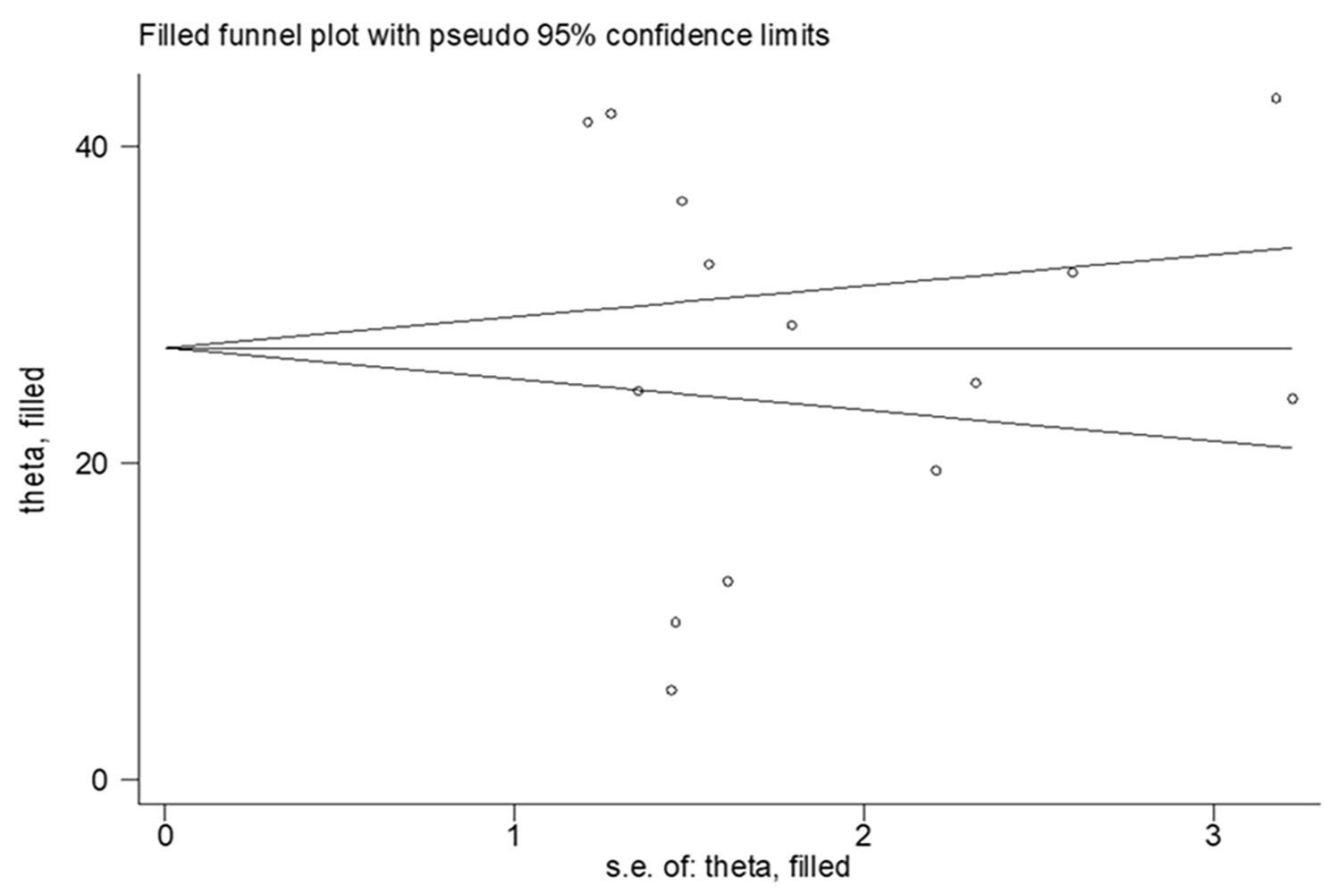
Trim and fill analysis for correcting publication bias of 14 studies

The pooled magnitude was changed from [16.39% (95% CI, 13.89%, 18.88%)] to [16.39% (13.23%, 19.42%)] after trim and fill analysis. The egger test only detected minimal publication bias; however, since the later confidence interval includes the first pooled size, there is no conspicuous difference between them which affects the final effect sizes **(**Fig. 5**)**.

### Predictors of mortality among cervical cancer patients in Ethiopia

After thoroughly literature review, we selected four commonly significant predictors of mortality among cervical cancer patients based on the frequency of being considered by different studies. Late diagnosed [RR = 2.65, 95% CI: (2.16, 3.26)], radiation therapy only [RR = 2.21, 95% CI: 1.65, 2.96], and being anemic [RR = 3.20, 95% CI: 1.73, 5.91] are the most common aggregate predictors of mortality for cervical cancer patients. Each of them was discussed by more than three studies.

**Fig. 6:**
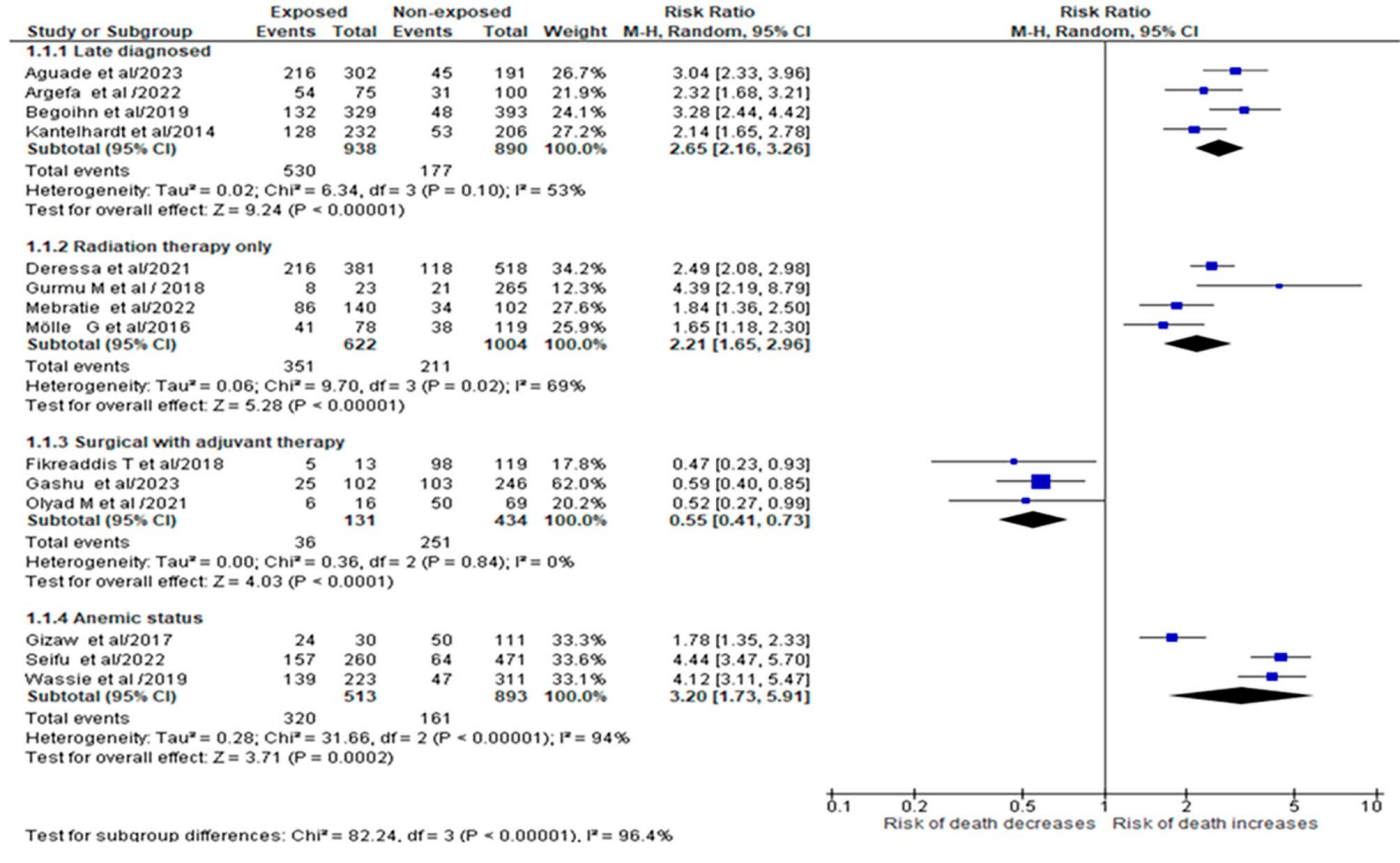
Forest plot briefing predictors of cervical cancer related mortality in Ethiopia

Another goal was to realize how recombinant therapy could decrease cervical cancer related mortality and improve survival rate of the patients had been identified from four studies conducted on survival of patients with cervical cancer. Survival status of cervical cancer patients can be increased as a result of surgery with adjuvant therapy when compared with cervical cancer patients treated by radiation therapy only [RR = 0.55, 95% CI: (0.41, 0.73)]. Thus, the output of our Meta analysis forwarded that 45% of mortality from cervical cancer can be averted by combination therapy than non-recombinant therapy (Fig. 6).

## Discussion

It was crucial to carry out a systematic review and meta-analysis in order to offer national strategic designers of the health sector advice. Despite the development of new cervical cancer therapies, significant mortality rates are still reported in low and middle-income countries [34, 35]. This could be due to the use of non-preferred methods and the limitations of care provisions, as well as the inability of patients in developing countries to cover the cost of therapy [34].

We carried out this review using data from 14 studies which included 9,260 cervical cancer patients. In accordance with the current meta-analysis, the pooled magnitude of cervical cancer patients’ mortality was reported as 16.39% with 95% confidence interval. This finding is consistent with the findings of a study done in America among non-Hispanic African American and white women, which reported a 14.2% death rate and a 13.3% review of LMIC, but more than the 5.49% found in urban China studies [5] and the two studies conducted in USA 7.9–11.9% and 4% respectively [35]. However our finding is less than review conducted in Thailand Bhutan 22.7% [36] and interventional study done in India which reports 25.32% died in the chemotherapy plus surgery group and 25.24% died in the concomitant chemotherapy plus radiotherapy group [37]. The reasons of this variation might be the difference between study design, characteristics of study population, treatment modalities and sociodemographic characteristics of the patients.

Among the common predictors of cervical cancer related mortality, late diagnosis of the disease was identified by this review. Four articles in which late diagnosis commonly discussed as the predictor were selected and analyzed, as seen from the above forest plot. From this finding, the risk of dying among late diagnosed cervical cancer patients were 2.65 times more likely when compared to those diagnosed early. The reason might be lack of cervical cancer screening awareness in developing countries increases progression of pre-cervical cancer lesion into invasive malignant stages which can metastasis to vital organs and have poor prognosis of the diseases, thus increases cervical cancer related mortality [38]. Furthermore, evidence of this suggests that five year survival rate for early diagnosed cervical cancer is 70% when compared to stage IIB to stage IV in which they have less than 20% survival rate [39]. As cancer stage increases, malignant metastasis also increase, which leads to compromising patient’s immunity to resist other infections, difficulty of treating the cases and these factors in aggregate increases the probability of death. This determinant was also reported by different literatures [40, 41].

This study also measured the contribution of combination of surgery with adjuvant therapy to alleviate cervical cancer related mortality. According to the pooled protective effect of recombinant therapy from these studies, 45% of cervical cancer related mortality can be averted by surgery with adjuvant therapy modality. The reason for this could be that, while surgical therapy is determined by tumour size and cancer stage, different evidence strongly suggests surgery to reduce tumour size and remove partial if all impossible. Adjuvant therapy is then used to reduce the rate of anaplasia and thus the cancer cell’s invasive capacity [42, 43].

The reports of four studies focused on contribution of radiation therapy only treatment modality to cervical cancer related mortality also selected in this review. The reports of those studies were examined at 95% confidence level. Thus the odds of cervical cancer related mortality among patient treated by radiation alone were 2 times more likely when compared with the patient treated by other treatment modalities. The reason for this could be that immunotherapy can achieve a synergistic effect with radiotherapy by directly inducing immunogenic death of tumour cells [44] reestablishing tumour vessels and regulating tumour cell phenotype [45], allowing immune cell infiltration and systemic therapeutic drug local infiltration [42, 46]. Acute and Long term toxicity of radiation therapy alone also supported by another study [47].

Being anemic was identified as the fourth most predictor of the cervical cancer related mortality from the four studies. The risk of death by cervical cancer patient increases three times as compared to normal hematocrit patients. This result implies that the risk of dying from cervical cancer was 3.2 times higher in patients with anaemia than in the control group. This could be because low hemoglobin levels in the blood cause oxygen deprivation in both cancerous and normal cells, resulting in tissue necrosis and exacerbating malignancy and cancer cell invasive capacity [48, 49]. The current finding is supported by the studies conducted in Canada [50] and Japan [51]. Synergic effect of anemia to cervical cancer related mortality is so preventable with effective implementation of its managements [52]. However, in this meta-analysis, anemia was highly significant to increase cervical cancer related mortality, but correction of anemia is so first priority to improve survival of cervical cancer patients while initiation of direct cancer patient therapy [53].

## Conclusion

The finding of this systematic review and meta-analysis reported high cervical cancer-related mortality at national level. Cervical cancer mortality was found to be strongly linked with late diagnosis and anaemia. However, this meta-analysis found that combining surgery with adjuvant therapy was associated with a significant decrease in cervical cancer-related mortality. This review also discovered that radiation therapy alone without combination therapy is less effective to improve cervical cancer patient survival than recombinant chemotherapy.

The report of our meta-analysis recommends that early diagnosis of tumor stage through cervical cancer screening program improves survival of cervical cancer. Furthermore, surgery with adjuvant therapy more encouraged to increase survival rate of cervical cancer patients. This meta-analysis disproves radiation therapy alone as a treatment modality for cervical cancer, as it did not improve patient survival. Accordingly, this finding supports the use of combination treatment modalities for cervical cancer. It is expected that future research better focus on comorbid treatment of cervical cancer patients to increase drug efficacy and to develop better treatment modalities.

### Limitation of the study

Most of the studies conducted in developing countries are non-randomized, as the cost-effectiveness of the studies for resources is limited. Thus, this meta-analysis included only non-randomized studies. Therefore, in the future, researchers should better focus on laboratory-based studies to identify predictors of cervical cancer-related mortality.

### Electronic supplementary material

Below is the link to the electronic supplementary material.

Supplementary Material 1

Supplementary Material 2

Supplementary Material 3

Supplementary Material 4

Supplementary Material 5

## Data Availability

The datasets used and/or analyzed during the current study are available freely and openly accessed in this article is supplemented.

## Abbreviations

AFT: Accelerated failure time
CIN: Cervical intraepithelial neoplastic
HIV: Human immunodeficiency virus
HPV: Human papillomavirus infection
CI: Confidence interval
I^2^: Inverse variance
OR: Odds ratio
PRISMA: Preferred Reporting Items for Systematic Reviews and Meta-analysis
SE: Standard error
SNNPR: Southern Nations and Nationalities of Ethiopia
WHO: World Health Organization
