# Supplementary material for: Common predictors of cervical cancer related mortality in Ethiopia. A systematic review and meta-analysis
Source: BMC Public Health. 2024 Mar 19;24:852. doi: 10.1186/s12889-024-18238-x (PMC10953061; doi:10.1186/s12889-024-18238-x)
Supplement: Supplementary file 2 — Supplementary Material 2 [file 12889_2024_18238_MOESM2_ESM.docx]

**Additional file2 text: Search strategy and data sources**

**Google Scholar**

(Cervical cancer OR survival of cervical cancer OR predictors of cervical cancer OR factors of cervical cancer AND mortality ), (cervical cancer treatment OR outcome of cervical cancer, cervical cancer mortality OR prevalence of cervical cancer mortality, factors of cervical cancer death OR death among cervical cancer OR women survival from cervical cancer), (Incidence of cervical cancer mortality OR determinants of cervical cancer death OR magnitude cervical cancer death OR/AND determinants AND cervical cancer mortality.

**PubMed**

#1 (“survival of cervical cancer” [All Fields] OR “survival of cervical cancer” [All Fields] OR “cervical cancer treatment” [All Fields] OR “Determinants of cervical cancer death” [All Fields]) AND (“women” [MeSH Terms] OR “patients” [MeSH Terms] OR “woman” [MeSH Terms]). #2 (“cervical cancer treatment outcome” [All Fields] OR (“prevalence of cervical cancer mortality” [MeSH Terms] OR (“cervical cancer” [All Fields] AND “radiation therapy” [All Fields]) OR predictors” [All Fields] OR “survival rate” [All Fields])) AND (“gynecological cancer” [MeSH Terms] OR “treatment modality” [MeSH Terms] OR “determinants of cervical cancer mortality” [MeSH Terms]). #3 #1 AND #2.

**EMBASE**

#1 ‘survival of cervical cancer /exp. OR ‘cervical cancer related mortality’ OR ‘low survival of cervical cancer’/exp. OR ‘survival status of cervical cancer’ OR ‘ART failure’/exp. OR ‘ART failure OR ‘ART failure’/exp. OR ‘adenoid hypertrophy’. #2 ‘treatment outcome of cervical cancer. #3 #1 AND #2. #4 #3 AND ([Gynecological cancer]/lim OR [cervical cancer realted mortality]/lim OR [HPV related death]/ lim OR [patient]/lim OR [women]/lim OR [predictors of cervical cancer death].

## **Cochrane library search strategy**

#1MeSH descriptor: [Cervical cancer] this term only

#2"Risk factors" or Predictors or factors (Word variations have been searched)

#3MeSH descriptor: [HPV infection] explode all trees

#4MeSH descriptor: [cervical cancer clinical stage] this term only

#5survival of cervical cancer or cervical cancer related death or low survival (Word variations have been searched)

#6 MeSH descriptor: [advanced cervical cancer stage] this term only

#7 incidence of cervical cancer mortality or cervical cancer death (Word variations have been searched)

#8 (#1 or #2 or #3) and (#4 or #5) and (#6 or #7)

#9 "HPV and cervical cancer" or "cervical cancer histopathology" (Word variations have been searched)

#10 (#1 or #2 or #3) and #9

#11 (#8 or #10)
