# Supplementary material for: Common predictors of cervical cancer related mortality in Ethiopia. A systematic review and meta-analysis
Source: BMC Public Health. 2024 Mar 19;24:852. doi: 10.1186/s12889-024-18238-x (PMC10953061; doi:10.1186/s12889-024-18238-x)
Supplement: Supplementary file 3 — Supplementary Material 3 [file 12889_2024_18238_MOESM3_ESM.docx]

**S5 Table. Quality assessment of included studies.**

| Cross-sectional and longitudinal studies | | | | | | | | | | | | | | | | | | |
| --- | --- | --- | --- | --- | --- | --- | --- | --- | --- | --- | --- | --- | --- | --- | --- | --- | --- | --- |
| Ref | Objective stated clearly | Population defined | Participation rate > 50% | Subjects from similar populations | Subjects from similar period | Inclusion criteria specified | Exclusion criteria specified | Sample size justification | Exposures measures before outcomes | Sufficient timeframe | Exposure levels examined | Exposure measures valid | Exposures assess more than once | 2003 / 2013 WHO outcome methods [1, 2] | Outcome assessors blinded | Loss-to-follow < 20% | Key potential confounding variables adjusted for | Quality |
| [3] | Yes | Yes | Yes | Yes | Yes | Yes | Yes | Yes | No | Yes | Yes | CD | No | CD | NR | NA | Yes | Good |
| [4] | Yes | Yes | Yes | Yes | Yes | NR | NR | No | No | Yes | Yes | Yes | Yes | Yes | NR | Yes | Yes | Good |
| [5] | Yes | Yes | Yes | No | Yes | NR | NR | Yes | No | Yes | Yes | Yes | No | Yes | NR | NA | Yes | Fair |
| [6] | Yes | Yes | Yes | Yes | Yes | Yes | Yes | NR | No | Yes | Yes | Yes | Yes | No | NR | Yes | Yes | Good |
| [7] | Yes | Yes | Yes | No | Yes | Yes | NR | Yes | No | Yes | Yes | Yes | No | No | NR | NA | No | Fair |
| [8] | Yes | Yes | Yes | Yes | Yes | Yes | Yes | Yes | No | Yes | Yes | Yes | No | CD | NR | NA | CD | Good |
| [9] | Yes | Yes | CD | Yes | Yes | Yes | Yes | No | Yes | Yes | Yes | Yes | No | CD | NR | NA | Yes | Good |
| [10] | Yes | Yes | NR | Yes | Yes | Yes | NA | NR | No | Yes | Yes | Yes | No | No | NR | NA | Yes | Fair |
| [11] | Yes | Yes | NR | Yes | Yes | NR | NR | NR | No | Yes | Yes | Yes | No | CD | NR | NA | CD | Fair |
| [12] | Yes | Yes | Yes | Yes | Yes | Yes | Yes | No | Yes | Yes | Yes | Yes | No | Yes | NR | NR | Yes | Good |
| [13] | Yes | Yes | CD | No | Yes | Yes | Yes | No | No | Yes | Yes | Yes | No | Yes | NR | NA | Yes | Fair |
| [14] | Yes | Yes | Yes | Yes | Yes | Yes | Yes | Yes | No | no | Yes | Yes | No | CD | NR | NA | CD | Fair |
| [15] | Yes | Yes | CD | Yes | Yes | Yes | Yes | No | Yes | Yes | Yes | Yes | No | CD | NR | NA | Yes | Good |
| [16] | Yes | Yes | NR | Yes | Yes | Yes | NA | NR | No | Yes | Yes | Yes | No | No | NR | NA | Yes | Good |

Abbreviations: CD - cannot determine; NA – not applicable; NR – not reported – RCT – randomized controlled trial; WHO – World Health Organization

# References

1. Satzke C, Turner P, Virolainen-Julkunen A, Adrian PV, Antonio M, Hare KM, Henao-Restrepo AM, Leach AJ, Klugman KP, Porter BD *et al*: **Standard method for detecting upper respiratory carriage of *Streptococcus pneumoniae*: updated recommendations from the World Health Organization Pneumococcal Carriage Working Group**. *Vaccine* 2013, **32**(1):165-179.

2. O'Brien KL, Nohynek H: **Report from a WHO Working Group: standard method for detecting upper respiratory carriage of *Streptococcus pneumoniae***. *Ped Infect Dis J* 2003, **22**(2):e1-11.

3. Aguade AE, Gashu C, Jegnaw T: **The trend of change in cervical tumor size and time to death of hospitalized patients in northwestern Ethiopia during 2018–2022: Retrospective study design**. *Health Science Reports* 2023, **6**(2):e1121.

4. Argefa TG, Roets L: **Malnutrition and the survival of cervical cancer patients: a prospective cohort study using the PG-SGA tool**. *Nutrition and Cancer* 2022, **74**(2):605-612.

5. Begoihn M, Mathewos A, Aynalem A, Wondemagegnehu T, Moelle U, Gizaw M, Wienke A, Thomssen C, Worku D, Addissie A: **Cervical cancer in Ethiopia–predictors of advanced stage and prolonged time to diagnosis**. *Infectious agents and cancer* 2019, **14**:1-7.

6. Deressa BT, Assefa M, Tafesse E, Kantelhardt EJ, Soldatovic I, Cihoric N, Rauch D, Jemal A: **Contemporary treatment patterns and survival of cervical cancer patients in Ethiopia**. *BMC cancer* 2021, **21**(1):1-7.

7. Fikeraddis Tarik TGy, Boka dhugasa: **TREATMENT OUTCOME ANDASSOCIATED FACTORS AMONG CERVICAL CANCER PATIENTS TREATEDAT TIKURANBESA SPECIALIZSED HOSPITAL ADDIS ABABA, ETHIOPIA.A RETROSPECTIVE STUDY**. *Addis Ababa reposistory,Oncology* 2018.

8. Gashu C, Tasfa B, Alemu C, Kassa Y: **Assessing survival time of outpatients with cervical cancer: at the university of Gondar referral hospital using the Bayesian approach**. *BMC Women's Health* 2023, **23**(1):1-14.

9. Gizaw M, Addissie A, Getachew S, Ayele W, Mitiku I, Moelle U, Yusuf T, Begoihn M, Assefa M, Jemal A: **Cervical cancer patients presentation and survival in the only oncology referral hospital, Ethiopia: a retrospective cohort study**. *Infectious agents and cancer* 2017, **12**:1-7.

10. Gurmu SE: **Assessing survival time of women with cervical cancer using various parametric frailty models: a case study at Tikur anbessa specialized hospital, Addis Ababa, Ethiopia**. *Annals of Data Science* 2018, **5**(4):513-527.

11. Kantelhardt EJ, Moelle U, Begoihn M, Addissie A, Trocchi P, Yonas B, Hezkiel P, Stang A, Thomssen C, Vordermark D: **Cervical cancer in Ethiopia: survival of 1,059 patients who received oncologic therapy**. *The oncologist* 2014, **19**(7):727-734.

12. Mebratie AE, Moges NA, Meselu BT, Melesse MF: **Time to death from cervical cancer and predictors among cervical cancer patients in Felege Hiwot Comprehensive Specialized Hospital, North West Ethiopia: facility-based retrospective follow-up study**. *Plos one* 2022, **17**(6):e0269576.

13. Mölle U: **Radiotherapeutische Behandlung des Zervixkarzinoms mit Cobalt-60 in Äthiopien**. University Hospital Tübingen; 2016.

14. Olyad Mose GM, Abebaw Fekadu,Mathewos Assefa: **FIVE-YEAR SURVIVAL OF WOMEN DIAGNOSED WITH CERVICAL CANCER AT TIKUR ANBESSA SPECIALIZED HOSPITAL, ADDIS ABABA, ETHIOPIA; A RETROSPECTIVE COHORT STUDY**. *Addis Ababa reposistory* 2021.

15. Seifu B, Fikru C, Yilma D, Tessema F: **Predictors of time to death among cervical cancer patients at Tikur Anbesa specialized hospital from 2014 to 2019: A survival analysis**. *PLoS One* 2022, **17**(2):e0264369.

16. Wassie M, Argaw Z, Tsige Y, Abebe M, Kisa S: **Survival status and associated factors of death among cervical cancer patients attending at Tikur Anbesa Specialized Hospital, Addis Ababa, Ethiopia: a retrospective cohort study**. *BMC cancer* 2019, **19**:1-11.
